# The role of actively open-minded thinking in willingness to take civic and political action on genome-edited food in the United States and Switzerland

**DOI:** 10.3389/fpsyg.2025.1565928

**Published:** 2025-06-02

**Authors:** Alex Segrè Cohen, Angela Bearth, Caitlin Drummond Otten

**Affiliations:** ^1^Center for Science Communication Research, School of Journalism and Communication, University of Oregon, Eugene, OR, United States; ^2^HF Partners, Zurich, Switzerland; ^3^School of Human Evolution and Social Change, Arizona State University, Tempe, AZ, United States

**Keywords:** genome editing, new genomic techniques, public perceptions of emerging technologies, actively open-minded thinking, civic engagement

## Abstract

Actively open-minded thinking (AOT) is a set of standards for good thinking, including avoiding overconfidence and a willingness to change one’s mind in response to new information. While AOT is theorized to aid individuals in navigating complex and polarizing issues, little prior literature has examined the role of AOT in public responses to emerging technologies. This study examines how engagement in AOT relates to civic and political action on genome editing. This controversial technology modifies plant genes for improved traits, offering transformative possibilities but bearing associated risks and uncertainties. We conducted surveys in the United States and Switzerland, two countries with different regulations toward genome-edited foods. We find inconsistent evidence for predictions between AOT and willingness to engage in actions supporting or opposing genome editing; further, in several cases, relationships varied by country. We discuss the implications for future research on AOT and public engagement in emerging technologies.

## 1 Introduction

Actively open-minded thinking is a cognitive style characterized by a willingness to seek out and carefully engage with evidence, including counter-attitudinal evidence, and change one’s opinion when the evidence warrants it (Baron, 2019; Baron et al., 2023; Haran et al., 2013; Stanovich and Toplak, 2023; Stanovich and West, 1997). Despite its promise as a skill that can aid individuals in navigating complex and polarizing issues, relatively little research has examined AOT’s role in shaping reactions to emerging technologies and global issues (e.g., Segrè Cohen et al., 2022; Árvai et al., 2023). We conducted a study to better understand the role of actively open-minded thinking (AOT) in attitudes and decisions regarding genome editing, an emerging technology in agriculture and foods. Genome editing is part of a broader array of New Genomic Techniques that can modify a plant’s genetic material to improve its traits (Chen et al., 2019). These techniques have the potential to improve food quality and safety; however, such food biotechnology has also been met with skepticism and opposition amongst segments of the public (e.g., Busch et al., 2021; Siegrist and Hartmann, 2020), making this topic a good case study for examining the role of AOT.

We report the results of consumer surveys in two countries with different regulatory environments for genome editing: the United States, where genome-edited foods are currently available on grocery store shelves, and Switzerland, where they are forbidden. This comparison enables us to better understand the role of AOT in public responses to genome editing across regulatory environments, which may shape perceptions and actions on emerging technologies (Bearth et al., 2024; Bearth and Siegrist, 2016). As our key outcome measures, we examine willingness to participate in civic (discussing with friends) and political (signing a petition, attending a public demonstration) actions on genome editing. From an applied perspective, individual engagement in civic and political actions is important to study because these actions represent bottom-up flows for collective action through which individuals can influence multi-level decision-making, for example, spurring firm- and government-level action (York et al., 2021). From a theoretical perspective, examining engagement in civic and political actions enables us to test theorized relationships between AOT and engagement in civic action that could promote reasoned and open-minded discussion on an issue, as well as political action that advocates for a particular policy alternative and may be reflective of closed-minded and insufficient thinking, or an outsourcing of thinking to domain experts (Baron et al., 2023).

Our study makes two key contributions: (1) we examine how AOT relates to willingness to engage in civic and political action on genome editing across regulatory contexts, furthering a theoretical understanding of the role of AOT in judgment and decision-making on emerging technologies; and (2) we present a translation of Baron’s AOT scale (Baron et al., 2023) into German for a Swiss sample, supporting future research examining AOT and other individual differences in non-U.S. populations. Below, we describe genome editing as the case study for our research before discussing prior research on AOT and participation in civic and political actions and how this prior research shaped the specific details of our study.

Genome editing involves using technologies such as CRISPR/Cas9 to modify a plant’s genetic material to improve its traits (Chen et al., 2019). Genome editing is one category of technologies referred to as New Genomic Techniques (NGTs), and it falls under the broad umbrella of gene technology. It is related to, but distinct from, other gene technologies such as genetic modification^1^. Unlike genetic modification, genome editing can but does not necessarily involve introducing foreign genes from another species into a plant. Instead, genome editing allows scientists to make targeted edits in the plant’s existing genome to engender desired changes in the plant. Like the older forms of genetic modification technology, genome editing can help food systems address threats: in agriculture, genome editing technology is being developed to address goals including improving crop yields, boosting crop quality (e.g., nutritional content), and increasing crop resistance to insects, fungi, bacteria, and viruses (Chen et al., 2019).

However, public opinion in both the United States and Switzerland is divided on agricultural gene technologies. A 2019 survey found that over half of American adults believe that genetically modified foods are fairly or very likely to increase the global food supply and lead to lower prices, but about half of American adults also believe that genetically modified foods are worse for health than non-genetically modified foods (Funk, 2020). In Switzerland, a 2021 survey found that a majority of Swiss citizens support extending the moratorium on genetic technology but also view some potential benefits of genome-edited crops as positive (Gfs.Bern, 2021).

Prior research on public reactions to emerging technologies, and gene technologies specifically, has often examined variables including affective reactions and risk and benefit perceptions (Bearth and Siegrist, 2016; Connor and Siegrist, 2010; Finucane et al., 2000; Scott et al., 2016; Siegrist and Hartmann, 2020; Slovic et al., 2007). This prior research found that greater acceptance of agricultural applications of gene technologies is positively associated with perceived benefits and negatively associated with perceived risks (Connor and Siegrist, 2010; Bearth and Siegrist, 2016). Siegrist and Hartmann (2020) found that factors influencing attitudes toward novel food technologies include the extent to which decision-makers trust food producers and regulators (with greater trust predicting greater acceptance). However, little prior literature has examined the role of AOT in public responses to emerging technologies. In contrast to intelligence or factual knowledge, AOT is considered a thinking disposition, or cognitive style, related to how people reason about evidence and make judgments (Stanovich and Toplak, 2023). Baron (1993, 2019) suggests that actively open-minded thinking encompasses critically engaging with problems by seeking out and carefully considering belief-inconsistent evidence, updating one’s beliefs to consider new evidence, and avoiding overconfidence in one’s favored conclusions. AOT is typically measured by asking people to indicate the extent to which they agree or disagree with statements like, ‘Changing your mind is a sign of weakness” (reverse-coded) and “People should always take into consideration evidence that goes against their beliefs” (Stanovich and West, 1997; Stanovich and Toplak, 2023). In Baron et al. (2023)‘s AOT scale, used here, individual differences in measured AOT are thought to reflect two key dimensions: promoting open-mindedness through avoiding myside bias, in which people search for and interpret evidence in ways that support their prior beliefs and convictions, and avoiding overconfidence in preferred conclusions. AOT is thus theorized to aid people in navigating the complex and uncertain informational landscape surrounding complicated and polarizing issues such as genome editing.

To better understand how AOT relates to judgments and decisions on genome editing, we examined willingness to engage in civic and political actions as our main dependent measures. Prior theory makes a distinction between civic engagement, which comprises voluntary actions seeking to engage with others and build community without directly seeking to influence public policy (e.g., volunteering, participating in organizations), and political engagement, which comprises activities that do directly seek to influence policy, politics and governmental institutions (e.g., communicating with public officials, voting) (e.g., Barrett and Brunton-Smith, 2017; Ekman and Amnå, 2012; Gil de Zúñiga et al., 2010; Hanus et al., 2018; Theiss-Morse and Hibbing, 2005; Wicks et al., 2014). In our study, we examined willingness to participate in the civic action of talking to a friend about genome editing and in the political actions of signing a petition or participating in a public demonstration. While talking to a friend need not have the goal of influencing policy and politics, and thus, in the theoretical framework we adopt for this paper, constitutes a civic rather than political action, we note that prior research suggests that talking about political issues may encourage participation in other civic and political actions (Gil de Zúñiga et al., 2010; McCoy and Scully, 2002). As our two political actions, we chose to examine signing a petition because it is a widely utilized political action in both countries of investigation (e.g., Wright, 2016; Hanus et al., 2018), and we chose to examine participation in public demonstrations as a measure of public engagement in collective action (e.g., Cologna et al., 2021).

From an applied perspective, these behaviors are important to study because they represent cross-level flows for collective action through which individual citizens may influence multi-level decision-making (York et al., 2021). From a theoretical perspective, these behaviors are important to study because they enable testing of the theoretical predictions of AOT. Participation in civic action is thought to be one pathway through which individuals can become good citizens, promoting civic discussion and tolerance of others’ ideas (though in reality, such goals are rarely achieved; Theiss-Morse and Hibbing, 2005), consistent with Baron et al. (2023)‘s conceptualization of AOT as a moral virtue that supports democratic decision-making. In contrast, participation in political action, with the goal of influencing public policy toward favored alternatives, may reflect closed-mindedness that is inconsistent with AOT if favored alternatives are supported by insufficient or biased thinking. However, Baron et al. (2023) also note that, as AOT is thought of as a set of standards for good thinking, those higher in AOT may “outsource” their thinking on certain problems to domain experts who are thought to adhere to the standards of AOT. Consistent with this conceptualization, Segrè Cohen et al. (2022); see also Árvai et al. (2023) found that higher AOT was associated with greater trust in experts during the COVID-19 pandemic. From this alternative perspective, participation in political action in support of policies suggested by the scientific consensus on genome editing may not reflect a closed mind and insufficient thinking inconsistent with AOT but rather an outsourcing of thinking to genome editing experts.

Our study extends prior work on AOT to assess its role in public reactions to emerging technologies, using genome edited food as a case study. We examined the relationship between AOT and willingness to engage in both civic and political actions *in support of* and *against* genome editing, making the following exploratory predictions. With respect to the relationship between AOT and willingness to engage in both civic and political actions, individuals higher in AOT are theoretically predicted to be more likely to thoughtfully evaluate the potential societal impacts of emerging technologies, and their openness to diverse perspectives may enable a more nuanced consideration of technological innovation’s societal implications. These tendencies may make them more likely to engage in conversations with others on genome editing, leading us to predict a positive relationship between AOT and willingness to discuss genome editing, both its benefits and concerns (EP1).

However, the prior literature offers mixed guidance about how actively open-minded thinking (AOT) relates to political actions such as signing petitions or participating in demonstrations. On one hand, AOT is associated with cognitive flexibility, willingness to revise beliefs, and epistemic humility (Baron et al., 2023), which may lead individuals to adopt more nuanced or ambivalent positions on controversial issues. From this perspective, individuals high in AOT might be less inclined to take part in collective political actions that often require strong convictions or clear positions, particularly when the issue at hand is scientifically or ethically complex, such as genome editing (EP2a).

On the other hand, recent research finds positive associations between AOT and risk perceptions, as well as trust in experts and compliance with expert behavioral recommendations, on a different issue, COVID-19 (Segrè Cohen et al., 2022; Árvai et al., 2023). If high-AOT individuals are more likely to view experts on genome editing as adhering to the principles of AOT and thus trustworthy, then high-AOT individuals may be more likely to engage in political action in a direction consistent with expert views on genome editing because they have “outsourced” their thinking to domain experts. Expert views and scientific consensus on genome editing and related techniques are largely positive and supportive of its usage (e.g., Gao, 2021; Klümper and Qaim, 2014); in early 2024, 37 Nobel Prize-winning scientists and over 1,500 researchers signed an open letter to Members of the European Parliament encouraging the loosening of regulations for New Genomic Techniques, citing the “unequivocal body of scientific evidence supporting” their usage and benefits (WePlanet, 2024). If high-AOT individuals adopt policy preferences in line with expert views on genome editing, we would thus predict that high-AOT individuals are more likely to participate in political actions *supporting* genome editing and less likely to participate in political actions *against* genome editing (EP2b). Table 1 provides an overview of the exploratory predictions made for the relationships between AOT and civic and political action on genome editing.

**Table 1:** Overview of exploratory predictions.

Number	Exploratory prediction
EP1	Positive relationship between AOT and willingness to discuss genome editing
EP2a	Negative relationship between AOT and willingness to participate in political actions
EP2b	Positive relationship between AOT and willingness to participate in political actions in support of genome editing Negative relationship between AOT and willingness to participate in political action against genome editing

Further, we examine the robustness of the observed relationships between AOT and civic and political action on genome editing across two countries: the United States and Switzerland. We selected these two countries for their substantively different regulations of and public attitudes toward genome editing in agriculture: in the United States, genetically modified and genome-edited crops are commonly found on store shelves, while in Switzerland, they are currently banned from being produced and imported, although an ongoing debate is taking place on the European and Swiss level to exempt certain types of genome editing from the existing moratorium. Additionally, previous research suggests that country-level differences, such as the structure of political institutions and the population size and composition, are related to civic and political engagement (Barrett and Brunton-Smith, 2017), further motivating our decision to compare engagement across the United States and Switzerland. According to a database maintained by the Carnegie Endowment for International Peace, public engagement in civic and political actions, from petition signing to (non)violent public demonstrations, is more common in the US than in Switzerland (Carnegie Endowment for International Peace, 2025). However, because genome editing in agriculture is illegal in Switzerland, but ongoing legislation may change this decision, civic and political actions (either in favor of or in opposition to genome editing) may be higher now than in previous years when the regulations were well-established. While public attitudes can certainly shape innovation and policy (Asioli et al., 2017; Rose et al., 2020), prior research suggests that the regulatory environment may also shape public attitudes toward emerging technologies (Bearth et al., 2024; Bearth and Siegrist, 2016), with stricter regulations signaling a potential risk, which might be associated with more negative public perceptions. This cross-country comparison enables us to rigorously test the theorized role of AOT in willingness to engage in civic and political action on AOT across cultural contexts, furthering the study of cross-country and cross-cultural differences in cognition and judgment and decision-making.

## 2 Materials and methods

### 2.1 Participants

We recruited adults over 18 who lived in the United States and German-speaking Switzerland to take a roughly 15-min survey. Participants were recruited from internet panels maintained by the market research firm Bilendi and respondi and sent an email with a link to the survey. Surveys were administered in English (United States) and German (Switzerland); the survey was developed in English, translated into German by a Swiss co-author (a native speaker of German), and then separately backtranslated into English. Within each country, quotas for age and gender were used to ensure that final samples had similar distributions of these variables relative to national statistics for each country.

The final sample comprised 1,202 participants, 613 from the United States and 589 from Switzerland. In the United States sample, the mean age was 46 (SD = 16), and 47% of participants identified as women, 51% identified as men, and 2% identified as non-binary, genderqueer, or a third gender; 25% reported holding low level of education (no high school, some high school, a high school diploma, or GED), 34% reported holding medium level of education (associate’s degree or some college), and 41% reported holding a high level of education (bachelor’s, graduate, professional degree). In the Swiss sample, the mean age was 46 (SD = 16), and 51% of participants identified as women, and 49% of the sample identified as men; 5% reported holding a low level of education (compulsory school), 58% reported holding medium level of education (vocational training, high school), and 35% reported holding a high level of education (university, applied university)^2^.

Ethical approval was received from the researchers’ institutions, and all participants provided informed consent.

### 2.2 Design and measures

The data used for this study was part of a larger questionnaire. Below, we describe the variables used for these analyses.

#### 2.2.1 Prior subjective knowledge of genome editing and plant breeding

Participants were asked to estimate how much they knew about genome editing in foods and agriculture and plant breeding in agriculture. Each was elicited on a 6-item scale (1 = I do not know what that is; 2 = almost nothing; 3 = very little; 4 = some; 5 = a fair amount; 6 = a great deal). Responses were averaged together to form an index measure of prior subjective knowledge (*M* = 2.8, SD = 1.2, *α* = 0.81).

#### 2.2.2 Baseline knowledge about genome editing and agricultural applications

Participants were next shown a short paragraph about genome editing to ensure that all had a similar baseline knowledge level about the topic. The paragraph described genome editing and how it might help address potential agricultural challenges and associated uncertainties. The paragraph also described how genome editing is distinct from genetic modification, based upon qualitative pretesting in which participants conflated the two technologies. The paragraph also mentioned some uncertainties and potential risks associated with the application of genome editing in agriculture (e.g., labeling and transparency, regulation). Next, participants were randomly assigned to read about one of three applications of genome-edited crops: we asked participants to read about specific applications of genome editing (i.e., disease-resistant potato, gluten-free wheat, cold-resistant soybean) to give them a more concrete context to reason about the risks, costs, and benefits of genome editing. We consulted with subject matter experts in molecular biology, plant breeding, policy, and regulation to ensure the initial description of genome editing and the three applications contained accurate information. A separate publication from this dataset tests for differences in perceptions and acceptance across the three applications (Bearth et al., 2024).

#### 2.2.3 Willingness to engage in civic and political action on genome editing

We measured participants’ willingness to engage in civic and political actions on genome editing, asking, “After hearing about genome editing and possible applications of genome editing in agriculture, how willing would you be to take the following actions?” Participants were asked about three actions supporting genome editing in agriculture and three opposing since not participating in actions supporting genome editing is not the same as participating in actions opposing genome editing. Responses were collected on scales from 1 to 7, where 1 = “very unlikely” and 7 = “very likely.” The two versions of these three actions were as follows: “Sign a petition for [against] genome editing in agriculture”; “Talk to a friend about the benefits of [your concerns about] genome editing in agriculture;” *and* “Participate in a public demonstration for [Publicly protest] genome-edited crops.”

#### 2.2.4 Actively open-minded thinking

Participants responded to a measure of actively open-minded thinking (AOT), the willingness to engage in critical thinking, seek out alternative points of view, and challenge and update one’s beliefs (Stanovich and Toplak, 2023), using a recently updated 11-item AOT scale from Baron et al. (2023). A contribution of our study is the translation of the AOT into German. Some small changes were made to the scale based on a qualitative pretest that was run with native German speakers from Switzerland (e.g., “Real experts” was changed to “Experts,” as the original terminology was unclear to the pre-testers). Scale items in English and German are in the Appendix Table 1.

#### 2.2.5 Covariates

We measured participants’ trust in institutions related to genome editing (subsequently referred to as trust in institutions) with an index measure consisting of three questions (*M* = 4.3, SD = 1.8, *α* = 0.90), each measured on a 7-item scale from 1 = strongly disagree to 7 = strongly agree: I trust government officials involved in regulating genome edited foods to make decisions that are safe for consumers; I trust companies involved in genome editing to be open and transparent about the uncertainties and benefits of foods created using genome editing; I trust scientific organizations to communicate openly and transparently about new findings on genome editing.

Participants were asked to provide demographic information, including gender, age, education, and political ideology. Participants were asked to select their gender from one of five options: “Man,” “Woman,” “Nonbinary, genderqueer, or gender nonconforming,” “Other,” or “Prefer not to answer.” We asked participants, “What is your age?” They inputted their age in years using only numbers. Education in the United States was measured like this: “What is the highest level of education you have completed?” and the participants were given the following options: “No high school,” “some high school,” “graduated high school/GED,” “some college,” “Associate’s degree,” “Bachelor’s degree,” “Graduate or Professional’s degree,” and “Prefer not to answer.” Education in Switzerland was measured with the same question but slightly adapted response options: “Mandatory school,” “apprenticeship,” “high school,” “higher degree or university,” “other,” and “prefer not to answer.”

Political ideology was measured by asking participants, “In terms of politics, do you think of yourself as,” and then they were provided with the following options: “very liberal,” “somewhat liberal,” “centrist,” “somewhat conservative,” “very conservative,” “not political,” and “prefer not to answer.” This measure was used in the United States and Switzerland, although these terms mean slightly different things in the two countries.

### 2.3 Analysis

We examined AOT scale performance across the two countries, looking at item functioning and internal consistency. We did not assess how responses differed across genome editing applications (see Bearth et al., 2024 for differences in consumer acceptance across applications). We conducted three mixed-design ANOVAs to examine differences in willingness to engage in the three civic and political actions we examined, in support of and against genome editing, across the United States and Switzerland. Direction (support *vs.* oppose) was treated as the within-subjects factor, and country (US *vs.* Switzerland) was treated as the between-subjects factor. We examined zero-order correlations between AOT, willingness to engage in civic and political actions, trust in institutions, and subjective knowledge of genome editing and plant breeding in both countries. We used linear regressions, conducted separately for each of the six actions, to test whether the relationship between willingness to engage and AOT differed across countries (United States or Switzerland), controlling for covariates and demographics. For the linear regression analyses, if we observed a statistically significant interaction between AOT and country (United States or Switzerland), we then used separate linear regressions, controlling for the same covariates, to examine the relationship between AOT and the outcome measure separately in each country.

## 3 Results

### 3.1 Actively open-minded thinking in the United States and Switzerland

We first examined American and Swiss participants’ responses to the Actively Open-Minded Thinking Scale. Compared to Swiss participants, American participants scored more highly, with a small effect size [Swiss *M* = 3.7, *SD* = 0.5 vs. United States *M* = 3.8, *SD* = 0.5; *t*(1200) = 2.9, *p* = 0.003, *d* = 0.17]. Scale reliability was α_English_ = 0.70 and α_Swiss_ = 0.62, lower than observed in previous research: Baron et al. (2023) report a Cronbach’s alpha of 0.86.

Some significant differences in responses across countries were observed, although effect sizes were small (see Table 2). An exception was the item “There is nothing wrong with being undecided about many issues” – Americans were more willing to agree to this statement than Swiss participants with a medium effect. The internal consistency analysis revealed that not all items performed well, particularly the item “Experts are willing to admit to themselves and others that they are uncertain or that they do not know the answer,” which was negatively correlated with the remaining scale in both countries. The item “There is nothing wrong with being undecided about many issues” was not correlated with the remaining scale in Switzerland.

**Table 2:** Means (M), standard deviations (SD), and corrected item-scale correlations for the Actively Open-Minded Thinking (AOT) scale in the United States of America and Switzerland.

AOT item	United States of America (*n* = 613)			Switzerland (*n* = 589)			*t*
	*M*	*SD*	*r_i_*	*M*	*SD*	*r_i_*	
Experts are willing to admit to themselves and others that they are uncertain or that they do not know the answer.	3.2	1.2	−0.08	3.0	1.2	−0.13	*t*(1200) = 2.9, *p* = 0.004, *d* = 0.17
People should take into consideration evidence that goes against conclusions they favor.	4.1	0.9	0.46	4.1	0.9	0.42	*t*(1200) = 1.0, *p* = 0.309, *d* = 0.06
Being undecided or unsure is the result of confused thinking. (R)	3.5	1.3	0.41	3.9	1.2	0.43	*t*(1199) = 4.7, *p* < 0.001, *d* = 0.27
People should revise their conclusions in response to relevant new information.	4.1	0.9	0.37	4.3	0.8	0.55	*t*(1200) = −5.3, *p* < 0.001, *d* = −0.30
Changing your mind is a sign of weakness. (R)	4.2	1.1	0.49	4.2	1.1	0.48	*t*(1200) = −0.2, *p* = 0.871, *d* = −0.01
People should search actively for reasons why they might be wrong.	3.8	0.9	0.24	3.8	1.0	0.24	*t*(1200) = 0.3, *p* = 0.735, *d* = 0.02
It is OK to ignore evidence against your established beliefs. (R)	3.8	1.2	0.44	3.8	1.2	0.45	*t*(1200) = 0.2, *p* = 0.871, *d* = 0.01
It is important to be loyal to your beliefs even when evidence is brought to bear against them. (R)	3.2	1.3	0.48	3.0	1.2	0.28	*t*(1199) = −3.2, *p* < 0.001, *d* = −0.18
There is nothing wrong with being undecided about many issues.	4.0	0.9	0.27	3.4	1.1	0.04	*t*(1199) = 10.0, *p* < 0.001, *d* = 0.58
When faced with a puzzling question, we should try to consider more than one possible answer before reaching a conclusion.	4.4	0.8	0.46	4.3	0.8	0.49	*t*(1200) = 2.2, *p* = 0.030, *d* = 0.13
It is best to be confident in a conclusion even when we have good reasons to question it. (R)	2.9	1.2	0.38	2.6	1.1	0.10	*t*(1200) = −5.5, *p* < 0.001, *d* = −0.32

AOT scale modified from Baron et al. (2023). M: mean; SD: standard deviation; r_i_: item-total correlation. (R) indicates reverse-scored for the final scale (original means and standard deviations are reported).

### 3.2 Willingness to engage in civic and political action on genome editing in the United States and Switzerland

We next examined the extent to which participants differed in their willingness to engage in civic and political action for and against genome editing across the United States and Switzerland (Table 3). We conducted three separate ANOVAs, one for each civic or political action, examining willingness to engage as a function of direction (in support of or against), country (the United States and Switzerland), and their interaction.

**Table 3:** ANOVAS indicating differences in willingness to engage in civic and political actions on genome editing in the United States and Switzerland.

Civic and political actions	USA (*n* = 613)	Switzerland (*n* = 589)
	*M (SD)*	*M (SD)*
*Talk to a friend*		
*For:* Talk to a friend about the benefits of genome editing in agriculture.	4.4 (1.9)^c^	4.1 (2.0)
*Against:* Talk to a friend about your concerns about genome editing in agriculture.	3.9 (1.9)	4.4 (1.9)
*Sign a petition*		
*For:* Sign a petition for genome editing in agriculture.	4.2 (1.9)^b, c^	3.5 (2.0)
*Against:* Sign a petition against genome editing in agriculture.	3.0 (1.9)	3.6 (2.0)
*Publicly protest*		
*For:* Participate in a public demonstration for genome edited crops.	3.5 (2.0)^a, b, c^	2.2 (1.7)
*Against:* Publicly protest genome edited crops.	2.6 (1.8)	2.4 (1.8)

M: mean; SD: standard deviation; ^a^ indicates significant differences between countries; ^b^ indicates significant differences between for and against actions; ^c^ indicates an interaction between country and action.

For the civic action of talking to a friend about the benefits or their concerns about genome editing in agriculture, a significant interaction effect was found [*F*(1, 1,200) = 47.2, *p* < 0.001, η^2^ = 0.04]. Swiss participants were more willing to talk to friends about their concerns than the benefits (*p* < 0.001). In contrast, the participants from the United States were more willing to talk to friends about the benefits of genome editing in agriculture (*p* < 0.001). Both main effects, for vs. against [*F*(1, 1,200) = 2.7, *p* = 0.103, η^2^ < 0.01] and country [*F*(1, 1,200) = 1.9, *p* = 0.167, η^2^ < 0.01] were not significant.

For the political action of signing a petition for or against genome editing in agriculture, a significant interaction effect was observed [*F*(1, 1,200) = 68.1, *p* < 0.001, η^2^ = 0.05]. Swiss participants reported a similar level of willingness to sign a petition for and against (*p* = 0.568). In contrast, participants from the United States were more willing to sign a petition for genome editing in agriculture (*p* < 0.001). There was also a significant main effect of for vs. against [*F*(1, 1,200) = 55.3, *p* < 0.001, η^2^ = 0.04] and a non-significant main effect of country [*F*(1, 1,200) = 0.7, *p* = 0.413, η^2^ < 0.01].

For the political action of participating in a public protest, there was a significant interaction effect [*F*(1, 1,200) = 83.4, *p* < 0.001, η^2^ = 0.07] and both main effects were significant [for vs. against: *F*(1, 1,200) = 40.4, *p* < 0.001, η^2^ = 0.03; country: *F*(1, 1,200) = 64.1, *p* < 0.001, η^2^ < 0.05]. The Swiss participants were equally willing or unwilling to protest for or against genome editing (*p* = 0.052), while the participants from the United States were more willing to protest for than against genome editing (*p* < 0.001).

### 3.3 Actively open-minded thinking and willingness to engage in civic and political action on genome editing in the United States and Switzerland

We next examined the relationship between AOT and willingness to engage in the six measured civic and political actions in the United States and Switzerland. Table 4 reports correlations between AOT, the six measured civic and political actions, trust in institutions, and prior subjective knowledge, separated by country.

**Table 4:** Correlations between willingness to engage in civic and political action on genome editing, trust, and actively open-minded thinking in the United States (*n* = 613) and Switzerland (*n* = 589).

	AOT	For: sign a petition	For: talk to a friend	For: participate in a public demonstration	Against: sign a petition	Against: talk to a friend	Against: participate in a public demonstration	Trust
United States of America								
AOT	–							
For: sign a petition	0.10*	–						
For: talk to a friend	0.11**	0.74***	–					
For: participate in a public demonstration	−0.10*	0.63***	0.59***	–				
Against: sign a petition	−0.31***	0.04	0.01	0.26***	–			
Against: talk to a friend	−0.12**	0.20***	0.31***	0.30***	0.59***	–		
Against: participate in a public demonstration	−0.32***	0.13**	0.13***	0.35***	0.73***	0.50***	*–*	
Trust in institutions	0.07	0.57***	0.51***	0.41***	−0.04	0.06	0.06	–
Prior subjective knowledge	−0.01	0.30***	0.33***	0.30***	0.13**	0.21***	0.20***	0.20***
Switzerland								
AOT	–							
For: sign a petition	0.01	–						
For: talk to a friend	0.16***	0.58***	–					
For: participate in a public demonstration	−0.27***	0.48***	0.36***	–				
Against: sign a petition	−0.16***	−0.01	−0.03	0.19***	–			
Against: talk to a friend	0.11*	0.12**	0.43***	0.12**	0.40***	–		
Against: participate in a public demonstration	−0.31***	0.06	0.02	0.47***	0.52***	0.29***	–	
Trust in institutions	0.17***	0.50***	0.51***	0.27***	−0.11*	0.10*	−0.05	–
Prior subjective knowledge	−0.09*	0.23***	0.26***	0.34***	0.15***	0.19***	0.27***	0.09*

*: *p* < 0.05, **: *p* < 0.01, ***: *p* < 0.001.

Although directions varied, we found that most actions were significantly related to AOT in both countries. In the United States, AOT was positively related to willingness to talk to a friend about the benefits of genome editing in agriculture and to sign a petition for genome editing in agriculture; AOT was negatively related to willingness to participate in a public demonstration for genome editing, willingness to sign a petition against genome editing in agriculture, willingness to talk to a friend about concerns about genome editing, and willingness to participate in a public demonstration against genome editing. In Switzerland, those with higher AOT were more willing to talk to a friend about both benefits and concerns regarding genome editing; AOT was unrelated to willingness to sign a petition for genome editing in agriculture and was negatively related to willingness to participate in a public demonstration for genome editing, willingness to sign a petition against genome editing in agriculture, and willingness to participate in a public demonstration against genome editing.

In the United States, AOT was unrelated to trust in institutions; in contrast, Swiss participants with higher AOT reported greater trust. In the United States and Switzerland, those with higher trust in institutions reported being more willing to take the three civic and political actions for genome editing. However, in the United States, trust in institutions was unrelated to the willingness to take action against genome editing, while in Switzerland, those with greater trust were more willing to talk to a friend about their concerns about genome editing and less willing to sign a petition against genome edited foods; trust was unrelated to the willingness to protest genome edited foods.

AOT was unrelated to prior subjective knowledge in the United States and negatively related to prior subjective knowledge in Switzerland. Across both countries, those with higher prior subjective knowledge were more likely to engage in all civic and political actions for and against genome editing.

We next conducted separate linear regression analyses for actions for and against genome editing in agriculture to assess whether the relationship between AOT and willingness to act differed across the two countries (Table 5). In the regression analyses, we controlled for the influence of the covariates of prior subjective knowledge, trust in institutions, political ideology, and demographics, namely age, gender, and education. If we observed a statistically significant interaction between AOT and country (United States or Switzerland), we then used separate linear regressions, controlling for the same covariates, to examine the relationship between AOT and the outcome measure separately in each country.

**Table 5:** Regressions willingness to engage in civic and political action on genome editing by country, actively open-minded thinking, and covariates.

	Civic and political actions in support of genome editing								
	Sign a petition, *R*^2^ = 0.34, *F*(9, 929) = 54.1, *p* < 0.001			Talk to a friend, *R*^2^ = 0.32, *F*(9, 929) = 48.2, *p* < 0.001			Participate in a public demonstration, *R*^2^ = 0.33, *F*(9, 929) = 50.2, *p* < 0.001		
	*B*	*t*	*p*	*B*	*t*	*p*	*B*	*t*	*p*
Constant	1.14	3.7	<0.001	0.90	2.9	0.003	1.28	4.1	<0.001
AOT	0.22	1.6	0.106	0.30	2.2	0.028	−0.41	−3.0	0.003
Country^1^	−0.79	−7.1	<0.001	−0.27	−2.5	0.013	−1.36	−12.3	<0.001
AOT * Country	−0.51	−2.3	0.020	0.07	0.3	0.756	−0.65	−3.0	0.003
Prior subjective knowledge	0.28	6.2	<0.001	0.39	8.6	<0.001	0.39	8.5	<0.001
Trust in institutions	0.57	18.2	<0.001	0.50	16.5	<0.001	0.36	11.4	<0.001
Age	0.00	0.1	0.950	0.00	1.1	0.282	−0.01	−1.8	0.068
Gender^2^	−0.11	−1.0	0.320	−0.06	−0.6	0.561	−0.18	−1.7	0.093
Education^3^	−0.05	−0.4	0.683	−0.11	−1.0	0.318	−0.05	−0.5	0.644
Political ideology^4^	−0.03	−0.6	0.524	0.06	1.3	0.203	−0.02	−0.3	0.751

	Civic and political actions in opposition to genome editing								
	Sign a petition, *R*^2^ = 0.13, *F*(9, 929) = 15.8, *p* < 0.001			Talk to a friend, *R*^2^ = 0.08, *F*(9, 929) = 9.3, *p* < 0.001			Participate in a public demonstration, *R*^2^ = 0.18, *F*(9, 929) = 22.1, *p* < 0.001		
	*B*	*t*	*p*	*B*	*t*	*p*	*B*	*t*	*p*
Constant	3.20	9.1	<0.001	2.61	7.3	<0.001	2.53	8.0	<0.001
AOT	−1.17	−7.5	<0.001	−0.48	−3.1	0.002	−1.23	−8.7	<0.001
Country^1^	0.35	2.8	0.005	0.57	4.5	<0.001	−0.38	−3.4	<0.001
AOT * Country	0.36	1.5	0.134	0.69	2.8	0.005	0.03	0.1	0.883
Prior subjective knowledge	0.26	5.0	<0.001	0.33	6.3	<0.001	0.29	6.2	<0.001
Trust in institutions	−0.11	−3.0	0.002	0.03	0.9	0.388	−0.01	−0.1	0.884
Age	0.01	1.4	0.171	0.00	1.1	0.294	0.00	−0.4	0.666
Gender^2^	−0.03	−0.3	0.796	0.04	0.3	0.729	−0.18	−1.6	0.104
Education^3^	−0.21	−1.7	0.087	0.04	0.3	0.771	−0.04	−0.4	0.720
Political ideology^4^	−0.16	−3.0	0.003	0.01	0.2	0.849	−0.15	−3.1	0.002

^1^ 0: United States of America, 1: Switzerland; ^2^0: man, 1: non-man; ^3^0: low, 1: high; ^4^ 1: very liberal – 6: very conservative.

First, we examined the actions *for* genome editing. For signing a petition supporting genome editing, we observed a statistically significant negative interaction between AOT and country, no main effect of AOT, and a significant negative effect of country. Separate regression analyses found a positive relationship between AOT and signing a petition in the United States (B = 0.27, 95% CI [0.01, 0.53], *p* = 0.045). In contrast, for Switzerland, no significant relationship was observed (B = −0.34, 95% CI [−0.71, 0.04], *p* = 0.079). There was no significant interaction effect for talking to a friend, but significant main effects of AOT and country were observed. Higher AOT and living in the United States were related to a higher willingness to talk to a friend about the benefits of genome editing. For participating in a public demonstration, we observed a statistically significant negative interaction effect between AOT and country and significant main effects of AOT (negative) and country (negative). In both countries, AOT was negatively related to willingness to participate in a public demonstration for genome editing. However, the effect was stronger in Switzerland (*β* = −0.46, 95% CI [−1.31, −0.72], *p* < 0.001) than in the United States (β = −0.37, 95% CI [−0.66, −0.08], *p* = 0.012).

Second, we examined the relationships with willingness to take action *against* genome editing. For signing a petition against genome editing, we observed no statistically significant interaction between AOT and country, a significant negative main effect of AOT, and a significant positive effect of country. Participants with higher AOT and participants from the United States expressed a lower willingness to sign a petition against genome editing. A significant positive interaction effect of AOT and country and significant main effects of AOT (negative) and country (positive) were observed for talking to a friend. In the United States, the relationship between AOT and willingness to talk to a friend about the risks of genome editing was negative (*B* = −0.46, 95% CI [−0.78, −0.15], *p* < 0.001), whereas this relationship was not significant in Switzerland (*B* = 0.18, 95% CI [−0.22, 0.59], *p* = 0.373). There was no significant interaction effect for participating in a public demonstration against genome editing, but significant main effects of AOT (negative) and country (negative) were observed. Participants with higher AOT and participants from Switzerland were less willing to participate in a public demonstration against genome editing.

Examining the covariates, we find that prior subjective knowledge was positively related to willingness to take action for all six civic and political actions we examined. Trust in institutions was positively related to willingness to engage in civic and political action for genome editing, unrelated to a willingness to talk to a friend or participate in a protest against genome editing, and negatively related to willingness to sign a petition against genome editing. Age, gender, and education were consistently unrelated to willingness to engage; more conservative respondents were less likely to report being willing to sign a petition or participate in a public demonstration against genome editing.

## 4 Discussion

Actively open-minded thinking (AOT), the willingness to challenge one’s beliefs and engage in reflective thought (Stanovich and Toplak, 2023), holds promise to aid individuals in navigating complex and polarizing issues. However, little research has examined AOT’s role in shaping reactions to emerging technologies and global issues (e.g., Segrè Cohen et al., 2022; Árvai et al., 2023). We report the results of a survey conducted in the United States of America and Switzerland, assessing the relationship between AOT and engagement in civic and political action on a novel and controversial emerging technology, genome editing in agriculture. Our findings further a theoretical understanding of the role of AOT in judgment and decision-making on controversial issues and contribute to a growing body of cross-cultural research examining how context may shape cognition and judgment and decision-making.

We predicted that individuals higher in AOT would be more willing to engage in the civic actions of talking to a friend about genome editing’s benefits and also about their concerns (EP1). Overall, our findings were partly consistent with EP1, as across both countries, individuals with higher AOT were more willing to talk to a friend about the benefits of genome editing. However, participants with higher AOT in the United States were unexpectedly less willing to talk to a friend about their concerns about genome editing, and AOT was unrelated to a willingness to discuss concerns in Switzerland after controlling for covariates and demographics. Our findings with respect to discussing genome editing are partially consistent with Baron et al. (2023)‘s conceptualization of AOT as a moral virtue that supports democratic decision-making, and with prior research finding that Twitter users with higher AOT generate longer tweets and are more likely to discuss broad and/or controversial topics like science, political ideology, discrimination, and religion (Carpenter et al., 2018).

Speculatively, the unexpected asymmetry we observe in communications about benefits *vs.* concerns regarding genome editing may stem from participants identifying different groups of friends with which to potentially discuss benefits and concerns. This pattern may reflect high-AOT individuals’ sensitivity to the anticipated *quality* of the conversation rather than a general avoidance of disagreement. High-AOT individuals may perceive benefit-focused discussions as more grounded in shared reasoning norms or less prone to derailment, particularly if benefits are framed in scientific or prosocial terms. Conversely, concern-focused discussions, especially in politically polarized contexts like the United States, may be seen as more likely to involve emotionally charged responses and reasoning styles inconsistent with the norms of open-minded thinking, thus leading to selective disengagement from those conversations. Related research on attitudes toward genetically modified foods and other controversial scientific issues such as vaccination finds that more extreme opponents of the scientific consensus know the least but think they know the most (Fernbach et al., 2019; Light et al., 2022), a pattern of perceptions opposite that of AOT. Thus, the asymmetry may reflect a preference not for engagement versus disengagement broadly, but rather for forms of engagement that are more conducive to reasoned, open-minded exchange.

We also predicted that individuals higher in AOT would be either less willing overall to engage in the political actions of signing a petition and participating in a public demonstration, reflecting an open-mindedness toward policy alternatives (EP2a), or more willing to engage in political actions in support of, but less willing to engage in political actions against, genome editing, reflecting the adoption of the views of genome editing experts, which are largely positive toward the technology (EP2b; e.g., Gao, 2021; Klümper and Qaim, 2014). Our findings regarding EP2 were mixed. Specifically, we found that AOT was negatively related to willingness to sign a petition against genome editing and to participate in a public demonstration for and against genome editing, consistent with EP2a. However, we found that those higher AOT from the United States were more willing to sign a petition for genome editing; in Switzerland, AOT was unrelated to willingness to sign a petition for genome editing. Our findings are largely inconsistent with prior research suggesting that critical thinking and open-mindedness may be positively, not negatively, related to political participation (Guyton, 1988; Sinatra et al., 2012).

Theoretically, involvement in political actions to influence public policy in favor of preferred alternatives could indicate a lack of open-mindedness inconsistent with AOT if these favored choices are grounded in inadequate rationale. As Baron et al. (2023) note, “the most dangerous political beliefs are those that are held with great confidence despite minimal or biased thinking” (p.1). However, the principles upheld by AOT also align with the notion that seeking assistance from reliable sources (e.g., outsourcing thinking to domain experts) can enhance decision-making processes by alleviating the cognitive burden on individuals, thereby facilitating the attainment of sound decisions without solely relying on personal judgment (Baron, 2019; Baron et al., 2023). In our previous research examining the relationship between AOT and public responses to COVID-19 (Segrè Cohen et al., 2022; Árvai et al., 2023), we found that high-AOT individuals reported greater trust in public health experts and were more likely to act in compliance with recommended behaviors, suggestive of outsourcing. In this study, across both the US and Switzerland, we find that those with higher trust reported being more willing to take the three civic and political actions for genome editing, consistent with substantial literature asserting that trust in institutions is positively associated with acting in line with scientific consensus (Algan et al., 2021; Pagliaro et al., 2021; Cologna and Siegrist, 2020).

There are several potential reasons why we did not consistently observe that high-AOT individuals were more likely to participate in political action for and less likely to participate in political action against genome editing (EP2b). While we find that high-AOT individuals report more trust in genome editing institutions in Switzerland, consistent with the “outsourcing” prediction of EP2b, in the United States, AOT and trust were uncorrelated. Our other work on COVID-19 (Segrè Cohen et al., 2022; Árvai et al., 2023) measured trust in experts as “public health experts,” while this present research measured trust via a three-item index of trust in governments, industry, and scientific organizations. The literature on social trust indicates that when individuals perceive institutions to have similar values to them (via salient value similarity), they are more likely to trust these institutions and use the information these institutions communicate to determine risk and benefit perceptions (Siegrist et al., 2000). However, because people may differentially trust different institutions (i.e., trusting government but not industry), trust in different actors may be differentially associated with both AOT and political actions for or against genome editing. For example, Cologna et al. (2021) found that Swiss students participating in Fridays for Future, a strike for increased government action on climate change, had higher trust in scientists and lower trust in governments compared to those who did not participate. The composite trust variable in our research had an alpha of 0.90; future research should measure trust in specific institutions with more nuanced scales (rather than one item per institution).

Additionally, the role of trust in institutions on perceptions of emerging technologies may vary when institutions engaged in producing and regulating that technology are not acting consistently. For example, in the Swiss context, leading scientists have signed a petition supporting New Genomic Techniques (WePlanet, 2024), but the government currently bans the technology. Research is needed to differentiate how actively open-minded thinkers perceive different institutions and ascertain *which* experts they outsource their information from. Future research should measure trust in different institutions separately and investigate the extent to which participants perceive those institutions as engaging in AOT. Future research should also extend these analyses to additional topics and emerging technologies in different regulatory contexts.

We report the results of a cross-country survey in the United States, where genome-edited foods are currently allowed on grocery store shelves, and Switzerland, where genome-edited foods are forbidden. Prior research has emphasized the importance of conducting research across countries and cultures and of studying non-WEIRD (Western, Educated, Industrialized, Rich, and Democratic) societies for building a scientific understanding of human cognition, motivation, and behavior (e.g., Barrett, 2020; Henrich et al., 2010; Medin et al., 2017). We note that, even across the two WEIRD societies we studied, we still observed substantive differences in the relationship between AOT and willingness to engage in civic and political action on genome editing, specifically, for half of the dependent measures we examined (three out of six), we observed a statistically significant interaction such that the relationship between AOT and the dependent measure differed across countries. Prior research suggests that regulatory environments may shape perceptions and actions on emerging technologies, including genome editing (Bearth et al., 2024; Bearth and Siegrist, 2016), such that stricter regulations are associated with lower consumer acceptance. Speculatively, different regulatory environments may also possess different informational environments, giving rise to differential associations between AOT and behavior in the United States and Switzerland. Given the potential for substantive differences in regulatory environments, cross-cultural studies may be particularly important for future research on controversial and emerging technologies.

We contribute to this future research by translating the AOT scale (Baron et al., 2023) into German for usage in the Swiss population. However, our translation of the AOT scale also suggests some important potential limitations. The reliabilities of the AOT scale in both the United States and Swiss samples were lower than expected from prior research, and item-level analyses suggest that some items performed poorly, possessing low correlations with the rest of the scale. Low reliability constrains how strongly the measure can correlate with other variables, impacting our ability to estimate associations between AOT and other variables (John and Benet-Martinez, 2000). Further, our measure of AOT relied on participants self-reporting their endorsement of AOT principles. Future research should develop reliable and culturally appropriate AOT measures to further investigate the relationships identified here.

Relatedly, we measured behavioral intentions rather than actual civic and political actions. To improve on the ecological validity of the findings, future research should use measures of actual behavior, given the well-documented attitude-behavior gap separating behavioral intentions from action on global issues such as climate change (e.g., Kollmuss and Agyeman, 2002; Whitmarsh et al., 2021).

With respect specifically to translating individual different measures, future research should delve further into the cultural dimensions of psychological constructs and traits, including AOT (e.g., Church, 2016). Our study did not explicitly address the potential cultural nuances of AOT or critically assess whether it is a universally applicable cognitive style. While we carefully pre-tested our translation of the AOT scale and changed its wording as necessary, our analyses here assume the validity of the extant items for measuring AOT in the Swiss context. Item-level analyses suggested differences in responses across Swiss and American participants, though they were primarily associated with small effect sizes. Future work could involve developing culturally informed measures or exploring alternative measures that better capture the cognitive processes relevant to AOT within specific cultural contexts. By doing so, researchers can ensure that their assessments align with the diverse ways individuals from various cultures approach and engage with information and ideas.

## 5 Conclusion

This study contributes to the growing body of literature on actively open-minded thinking (AOT) and its implications for public engagement with emerging technologies. Our findings shed light on the complex relationship between AOT and civic and political action regarding genome editing, a controversial and rapidly advancing field with transformative potential for agriculture and nutrition. While AOT has been conceptualized as a critical component of good thinking, our study reveals inconsistent evidence for its relationship to public engagement with genome editing. Furthermore, the varying associations observed between AOT and civic and political actions across different countries underscore the importance of considering contextual factors in understanding public responses to emerging technologies.

Empirical social science research analyzing public attitudes toward and understanding of emerging technologies such as genome editing is needed to inform science communications that aid people in making decisions consistent with their values (e.g., Árvai, 2014; Fischhoff, 2013; Wong-Parodi et al., 2016). Despite the challenges posed by polarizing topics and conflicting sources of information, fostering critical thinking skills among the public may enhance trust in legitimate experts and scientific consensus (Segrè Cohen et al., 2022; Árvai et al., 2023) and holds promise for improving decision-making. As democratic societies continue to grapple with the implications of emerging technologies, future research must advance scientific understanding of public engagement in civic and political action on emerging technologies and other pressing societal issues. Understanding *how* and *why* individuals choose to participate and the underlying traits or skills these individuals may have can inform research and policy aimed at improving collective democratic decision-making efforts. By addressing these gaps in scientific knowledge, research can aid policymakers, scientists, and communicators in navigating the complexities of public opinion and fostering informed decision-making on science and technology.

## Data Availability

The datasets generated for this study, as well as supplemental material can be found in Open Science Framework https://osf.io/k7hj6/?view_only=e8f0700959a345b089d929d9b4595f1c.

## Footnotes

^1^We note here that different stakeholders may use different terminology and make different distinctions across types of gene technologies.

^2^The classification scheme followed international standards set by the OECD and ISCED (International Standard Classification of Education), which account for structural differences among countries. In the United States, earning a high school diploma or GED usually signifies the completion of upper secondary education and is not often associated with vocational qualifications; therefore, it is viewed as a low level of education unless accompanied by additional postsecondary training. Conversely, upper secondary education in Switzerland frequently includes formal vocational training (such as via the dual-track apprenticeship system), allowing individuals to directly enter skilled professions directly. As a result of incorporating vocational credentials at the upper secondary level, this category is classified as a medium level of education according to OECD/ISCED standards.
