# Supplementary material for: The role of actively open-minded thinking in willingness to take civic and political action on genome-edited food in the United States and Switzerland
Source: Front Psychol. 2025 Jun 2;16:1565928. doi: 10.3389/fpsyg.2025.1565928 (PMC12169248; doi:10.3389/fpsyg.2025.1565928)
Supplement: Supplementary file 1 [file Table_1.docx]

Appendix Table 1. Actively Open-Minded Thinking Scale Items (Baron et al, 2023) in the original English, and translated into German

| **Original Item in English** | **German Translation** |
| --- | --- |
| Experts are willing to admit to themselves and others that they are uncertain or that they don’t know the answer. | Experten sind bereit, sich selbst und anderen gegenüber zuzugeben, dass sie unsicher sind oder die Antwort nicht kennen. |
| People should take into consideration evidence that goes against conclusions they favor. | Menschen sollten Erkenntnisse auch dann berücksichtigen, wenn diese den gewünschten Schlussfolgerungen widersprechen. |
| Being undecided or unsure is the result of confused thinking.* | Wenn man unentschlossen ist, ist dies ein Zeichen dafür, dass man nicht klar denken kann. |
| People should revise their conclusions in response to relevant new information. | Man sollte bereit sein, seine Meinung zu überdenken, wenn man relevante neue Informationen erhält. |
| Changing your mind is a sign of weakness.* | Seine Meinung zu ändern ist ein Zeichen von Schwäche. |
| People should search actively for reasons why they might be wrong. | Menschen sollten aktiv nach Gründen suchen, warum sie falsch liegen könnten. |
| It is OK to ignore evidence against your established beliefs.* | Es ist in Ordnung, Gegenargumente nicht zu berücksichtigen. |
| It is important to be loyal to your beliefs even when evidence is brought to bear against them.* | Es ist wichtig, seinen Standpunkt zu halten, auch wenn es Argumente dagegen gibt. |
| There is nothing wrong with being undecided about many issues. | Es ist nicht schlimm, in vielen Fragen keine klare Position zu beziehen. |
| When faced with a puzzling question, we should try to consider more than one possible answer before reaching a conclusion. | Wenn man mit einem kniffligen Problem konfrontiert ist, sollte man mehrere Lösungen in Betracht ziehen, um zu einer Schlussfolgerung zu gelangen. |
| It is best to be confident in a conclusion even when we have good reasons to question it.* | Es ist wichtig einer Schlussfolgerung zu vertrauen, selbst wenn es gute Gründe gibt diese in Frage zu stellen. |

** indicates reverse-scored.*
